# Cerebral blood perfusion deficits using dynamic susceptibility contrast MRI with gadolinium chelates in rats with post-ischemic reperfusion without significant dynamic contrast-enhanced MRI-derived vessel permeabilities: A cautionary note

**DOI:** 10.1371/journal.pone.0201076

**Published:** 2018-07-25

**Authors:** Seokha Jin, MungSoo Kang, HyungJoon Cho

**Affiliations:** Department of Biomedical Engineering, Ulsan National Institute of Science and Technology, Ulsan, South Korea; Henry Ford Health System, UNITED STATES

## Abstract

In this study, we quantified perfusion deficits using dynamic susceptibility contrast magnetic resonance imaging (DSC-MRI) with an extravasating contrast agent (CA). We also investigated the efficacy of leakage compensation from CA pre-load in brains from post-ischemic rat models without significant dynamic contrast-enhanced MRI (DCE-MRI)-derived vessel wall permeability. DSC measurements were obtained using fast (0.3 s) echo-planar imaging in both normal rats and rats with transient middle carotid artery occlusion (MCAO) (1-h MCAO, 24-h reperfusion) after successive administrations of gadoterate meglumine (Dotarem) and intravascular superparamagnetic iron oxide nanoparticles (SPION). The relative cerebral blood volume (CBV) and cerebral blood flow (CBF) values acquired using Dotarem were significantly underestimated (~20%) when compared to those acquired using SPION in ipsilesional post-ischemic brain regions. A slight overestimation of relative mean transit time was observed. Areas with underestimated CBV and CBF values from the corresponding error maps encompassed the area of infarcted tissue (apparent diffusion coefficient < 500 μm^2^/s) and mostly coincided with the area wherein conspicuous longitudinal relaxation time differences were observed pre- vs. post-injection of Dotarem. The DSC measurements with significant pre-load (0.3 mmol·kg^-1^) of Dotarem displayed minimal perfusion deficits when compared to those determined using the reference intravascular SPION.

## Introduction

Post-ischemic reperfusion measurements in small animal models provide useful information for the optimization of intervention therapies and the evaluation of prognostic assessments [1,2]. As brain perfusion provides important information regarding the functional status of stroke lesions, several investigations have been carried out to develop accurate magnetic resonance (MR) measurements and delineation strategies for post-ischemic hypo- and hyper-perfused lesions [3,4]. Dynamic susceptibility contrast MR imaging (DSC-MRI) provides highly sensitive perfusion information and can be used to estimate the exogenous tracer concentration. Thus, it can also be used to extract information regarding cerebral perfusion, including cerebral blood flow (CBF), cerebral blood volume (CBV), and mean transit time (MTT), based on drug tracer models [5–7]. Typically, such pharmacokinetic models assume that the bolus-injected contrast agent (CA) does not extravasate and that the underlying blood vessels are randomly oriented. These are reasonable assumptions for a normal brain with an intact blood-brain barrier (BBB) when using gadoterate meglumine (Dotarem, Guerbet). However, when the BBB is disrupted (e.g., due to the presence of tumors or stroke), Dotarem, which is a member of the most commonly used family of MRI CAs, leaks through vessel walls. Consequently, in the presence of a damaged BBB, the DSC-MRI signal is significantly different from that calculated based on the no-leak assumption. Several studies have reported apparent DSC-MRI signal underestimations and correction methods for extravasating CAs used to image tumors with leaky vessels [8–13].

In studies of brains with early (<24 h) post-ischemic reperfusion and relatively small BBB leakage (insignificant *K*^*trans*^ obtained based on DCE-MRI [14]), the resulting DSC-MRI perfusion deficits are sometimes overlooked. In addition, the efficacy of leakage compensation using CA pre-load has not been directly quantified in a post-ischemic animal model. Systematic underestimations of cerebral perfusion parameters derived using DSC-MRI are problematic, especially for longitudinal animal studies, even if they are much smaller than those observed in cases of leaky tumors. This is because the DSC-MRI findings will result in ambiguity in the CBF and CBV threshold criteria used to distinguish the normal and hyper-perfused areas of the lesion. A previous study compared leaky vessel-derived CBF values obtained using DSC-MRI to those obtained using another MRI modality, namely, arterial spin labeling, in a stroke model [15]. However, these values were not compared to those obtained using an intravascular CA.

Accurate monitoring of first-pass (0–4 s in rat, as shown in S1 Fig) DSC time curves requires fast acquisition methods, such as echo-planar imaging (EPI), for application in animal models. This renders DSC-MRI signals much more sensitive to *T*_*1*_ and susceptibility changes due to CA leakage in animal model studies. Post-ischemic hyper-perfusion (~1 day) was frequently reported in the 1-hour middle cerebral artery occlusion (MCAO) animal model, which potentially has compromised BBB integrity in post-ischemic brain regions [3]. Therefore, a systematic comparison between dual DSC-MRI methods using extravasating and intravascular CAs in this model may be used to quantify and localize the perfusion errors and efficacy of CA pre-load in brains subjected to post-ischemic reperfusion.

In this study, an additional intravascular SPION measurement served as a reference to eliminate the confounding factor of vessel wall permeability following fast (repetition time [TR] = 0.3 s) DSC-MRI acquisition with EPI using Dotarem [13]. The DSC-MRI time curves obtained using either Dotarem or SPION were then directly compared for both normal rats and rats subjected to transient (1-hour) middle carotid artery occlusion (tMCAO) surgery following 1-day reperfusion. The cerebral blood perfusion underestimation errors were then quantified. The measured perfusion underestimation errors for both CAs and the apparent diffusion coefficient (ADC) values were used to segment the ipsi-stroke hemisphere. Cerebral perfusion errors, ADCs, and relative CBV values were compared among the infarct, peri-infarct, and normal zones. The efficacy of leakage compensation due to CA pre-load in brains from post-ischemic rat models was then studied using fast DSC-MRI acquisitions.

## Methods

### Animal preparation

The experiments were approved by the Institutional Animal Care and Use Committee of Ulsan National University of Science and Technology. Female Sprague-Dawley rats (SD; weight: 150–250 g) were obtained from Orient Bio (Gyeonggi, Republic of Korea). The SD rats were anesthetized with isoflurane during the MRI scan. To compare the two different DSC-MRI derived perfusion maps, rats in the normal (n = 3) and stroke (n = 6) groups were injected twice with the different CAs (Dotarem and SPION), as shown in S2 Fig. Two Dotarem injections were also performed in normal rats (*n* = 3). For both leakage compensation and vessel wall permeability estimations, DCE-MRI acquisition was performed before the two DSC-MRI acquisitions in the additional stroke group (n = 3). Rats in the stroke group were subjected to 1-h intraluminal monofilament (0.35 mm diameter filament, Doccol Corporation, USA) MCAO followed by 1-day reperfusion.

### Magnetic resonance imaging

All studies were performed using a 7-T MR scanner (Bruker, Germany) with a 40-mm volume coil and a surface coil. Dotarem and SPION were injected to evaluate the area under the curve (AUC) values of the DSC signals for the extravasating and intravascular CAs, respectively. AUC values for successive Dotarem-Dotarem injections were obtained in the control experiments using normal rats (n = 3). Corresponding values for successive Dotarem-SPION injections were also obtained (n = 3). For the tMCAO model, AUC values for successive Dotarem-SPION injections were obtained in rats subjected to stroke and reperfusion (n = 6). To identify regions of damaged BBB in the rats subjected to stroke, we obtained pre- and post-injection *T*_1_ maps. ADC maps and *T*_2_-weighted images were also obtained for rats subjected to stroke. All of the MRI procedures are detailed in S2 Fig. For both leakage compensation and vessel wall permeability estimations, the DCE-MRI acquisitions were performed before the two DSC-MRI acquisitions (Dotarem-SPION injections) in the additional stroke group (n = 3).

The DSC-MRI perfusion maps were acquired using a gradient-echo EPI sequence with the following pulse sequence parameters: TR = 300 ms, effective echo time (TE) = 17 ms, field of view (FOV) = 30 × 30 mm^2^, matrix size = 96 × 96, number of slices = 3, number of averages (NA) = 1, slice gap = 0.2 mm, slice thickness = 1 mm, bandwidth = 3.5 × 10^5^ Hz, number of segments = 1, flip angle = 35°, and temporal resolution = 0.3 s. The Dotarem and SPION injection doses were 0.3 mmol·kg^-1^ (130 μl) and 0.075 mmol·kg^-1^ (75 μl), respectively.

The *T*_*1*_ maps were obtained using rapid acquisition with relaxation enhancement (RARE) with variable TR (RAREVTR [16]) with the following parameters: TR = 80, 150, 200, 400, 800, 1200, 1600, 2000, 2500, 3000, and 4500 ms; RARE factor = 4; effective TE = 4.58 ms; NA = 1; FOV = 30 × 30 mm^2^; matrix size = 96 × 96; number of slices = 3; slice gap = 0.2 mm; and slice thickness = 1 mm.

The ADC maps were acquired using diffusion-weighted EPI with the following parameters: TR = 5000 ms; number of segments = 4; effective TE = 20 ms; b-values = 200, 400, 600, and 1000 s·mm^-2^; NA = 1; FOV = 30 × 30 mm^2^; matrix size = 96 × 96; number of slices = 3; slice gap = 0.2 mm; and slice thickness = 1 mm. Three ADC maps along the *x*, *y*, and *z* directions were averaged to obtain trace ADC values.

The *T*_*2*_-weighted images were obtained using RARE [17] with the following parameters: TR = 5000 ms, RARE factor = 4, effective TE = 30 ms, NA = 2, FOV = 30 × 30 mm^2^, matrix size = 256 × 256, number of slices = 20, slice gap = 0 mm, and slice thickness = 0.5 mm.

DCE-MRI data were acquired using fast low angle shot [18] with the following parameters: TR = 35 ms, TE = 1.9 ms, NA = 1, FOV = 30 × 30 mm^2^, matrix size = 96 × 96, number of slices = 3, slice thickness = 1 mm, number of repetitions = 180, temporal resolution = 3.36 s, and flip angle = 30°. The Dotarem injection dose for DCE-MRI was 0.1 mmol·kg^-1^. The Dotarem injection was followed by a 0.1–0.2 mmol·kg^-1^ flush (after 15 min) after the acquisition in studies of leakage compensation with CA pre-load for the subsequent fast DSC-MRI [13].

### Data analysis

For the *in vivo* study, two relative CBV (rCBV) maps for the first and second CA administrations were estimated from the AUC measurements from 0 to 4 s. The equation Δ*R*_2_*(t) $\left(=-\frac{1}{TE}ln\left(\frac{S_{post}(t)}{S_{pre}}\right)\right)$ should capture the first-passage of CA, as shown in S1 Fig. Both rCBV maps acquired before the normalization process are shown in S3 Fig. Because the two AUC maps contained relative values, the AUC map of the first administration (Dotarem) was normalized to that of the second administration (SPION) for the region with an intact BBB. For the normalization, the intact-BBB region was drawn manually on the right (normal group) or contralateral (stroke group) hemisphere. As shown in S3 Fig, scatterplots and linear fittings were performed for both AUC_Dotarem_ and AUC_SPION_ values for the intact-BBB region. The AUC_Dotarem_ map obtained after the first injection was then divided based on the slope of the regressed line for the intact-BBB region. The corresponding normalized AUC_Dotarem_ map (nAUC_Dotarem_) is referred to as rCBV_Dotarem_. Similarly, the AUC_SPION_ map is referred to as rCBV_SPION_. Following the above normalization procedure, the AUC ratio between the first and second injections was always 1 by definition (*y = x*) for intact-BBB regions, as shown in S3 Fig. Consequently, the rCBV_Dotarem_ values for the stroke region can be directly compared to those in the reference rCBV_SPION_. rCBF_SPION_, rCBF_Dotarem_, the relative MTT for SPION (rMTT_SPION_), and rMTT_Dotarem_ were also calculated. The rCBV_error_ map was computed by dividing the rCBV_SPION_ map by the rCBV_Dotarem_ map. We used a similar procedure to generate the rCBF_error_ and rMTT_error_ maps. For the statistical comparisons, a region of interest (ROI) was defined in the left hemisphere (normal group) or the BBB-disrupted region (stroke group). For the stroke group, the BBB-disrupted region was identified using the *T*_1_ difference map, which illustrates the difference between the *T*_1_ maps obtained before and after the Dotarem injection.

Voxel-wise ADC values (*S* = *S*0×*e^−ADC×b^*) were estimated for three gradient directions and averaged to obtain the ADC map. Areas of infarction (ADC < 500 μm^2^/s), peri-infarction with BBB damage (ADC > 500 μm^2^/s, rCBV_error_ > threshold), and normal tissue (ADC > 500 μm^2^/s, rCBV_error_ < threshold) were segmented by thresholding each value correspondingly in the ipsi-stroke hemisphere [19]. The threshold value was equal to (mean_rCBVerror_^contra.^ + std_rCBVerror_^contra.^), where mean_rCBVerror_^contra.^ and std_rCBVerror_^contra.^ were the average value and standard deviation of rCBV_error_ in the intact contralateral brain hemisphere, respectively. Histograms of the ADC, rCBV_error_, and rCBV_SPION_ (*V*_*p*_) values for the corresponding areas were then generated for use in the characterizations.

For the vessel wall permeability estimation, the DCE-MRI time curves were converted to Δ*R*_*1*_ values and fitted using the extended Toft model [20] to estimate *K*^*trans*^, *V*_*e*_, and *V*_*p*_. Pre- and post-injection *T*_*1*_ values were also sequentially measured in order to enable longitudinal monitoring of the leakage of Dotarem. The efficacy of leakage compensation using CA preload (0.1 mmol·kg^-1^ for DCE-MRI followed by a 0.1–0.2 mmol·kg^-1^ flush) was investigated by comparing the rCBV maps for the subsequent Dotarem- and SPION-derived DSC-MRI data.

## Results

### Extravasating (Dotarem) and intravascular (SPION) CAs in the 1-h MCAO 1-day reperfusion rat model

To evaluate the extent of extravasation for both Dotarem and SPION injections, *T*_1_ maps were measured before and after the CA injections. As shown in S4 Fig, the *T*_1_ values of the infarction region (ADC < 500 μm^2^/s) were significantly lower than those of the contralateral region (Wilcoxon rank sum test, p < 0.01, p = 0.001) after the Dotarem injection (0.3 mmol/kg) in the tMCAO model. In contrast, no significant differences were observed between the pre- and post-injection *T*_1_ maps (Wilcoxon rank sum test, p > 0.05, p = 0.229) after the SPION injection, indicating the absence of significant leakage of SPION at this time point. Note that the large-vessel region of the contralateral hemisphere was avoided when manually drawing the ROI because this region exhibited a shortened *T*_1_ value even though the CA was not extravasated. The same ROI was used to compare the Dotarem and SPION results. Statistical analysis of the differences in *T*_1_ measurements pre- vs. post-SPION injection confirmed that SPION did not extravasate to a noticeable degree in rats subjected to 1-h tMCAO and 1-day reperfusion, as shown in S4 Fig.

### Comparisons of DSC-MRI times curves obtained following Dotarem vs. SPION injections

**Fig 1:**
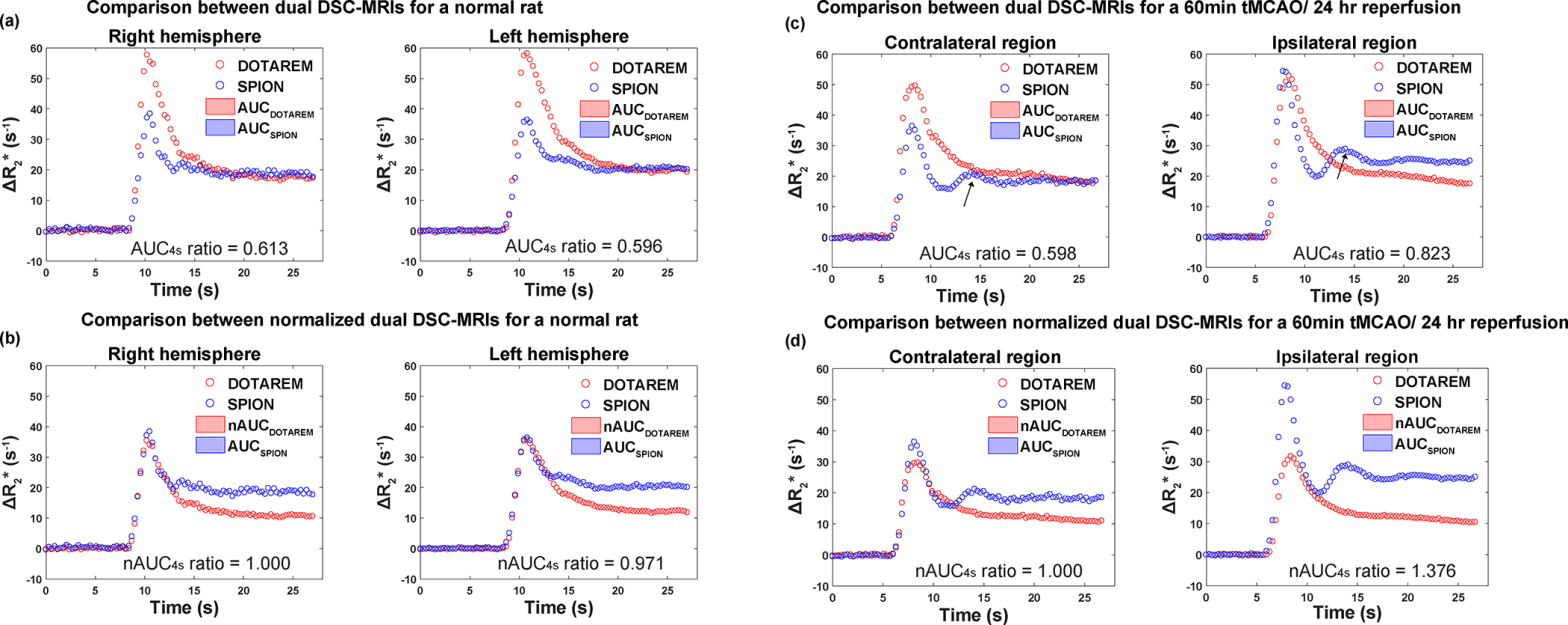
The process of nAUC ratio value estimation. (a) *ΔR*_2_* curve and AUC of SPION and Dotarem for unnormalized DSC-MRI signal of normal rat. Corresponding AUC ratio values were 0.613 and 0.596 for right and left hemispheres, respectively. (b) *ΔR*_2_* curve and AUC of SPION and Dotarem for normalized DSC-MRI signals of normal rat. Corresponding nAUC ratio values were 1 and 0.971 for right and left hemispheres, respectively. (c) *ΔR*_2_* curve and AUC of SPION and Dotarem for unnormalized DSC-MRI signal of tMCAO rat. Corresponding AUC ratio values were 0.598 and 0.823 for contralateral and ipsilateral hemisphere, respectively. (d) *ΔR*_2_* curve and AUC of SPION and Dotarem for normalized DSC-MRI signal of tMCAO rat. Corresponding nAUC ratio values were 1 and 1.376 for right and left hemispheres, respectively. Black arrows in (c) represent the second peak from recirculation from SPION DSC-MRI times curves.

The experimental DSC-MRI-derived Δ*R*_2_*(t) curves for rats in the normal and tMCAO groups are shown in Fig 1A and 1C, respectively. The black arrows in the SPION time curves in Fig 1C indicate the apparent second peak due to recirculation [6,21]. This peak was usually unobservable in the Dotarem time curves. After the normalization of AUC_Dotarem_ to AUC_SPION_, rCBV_Dotarem_ (nAUC_Dotarem_) was significantly smaller than rCBV_SPION_ (AUC_SPION_) for the ipsilesional hemisphere in the tMCAO model (Fig 1D). rCBV_error_ was thus greater than 1 for the BBB-disrupted model. No significant differences in rCBV_Dotarem_ were observed between the two hemispheres in rats in the normal group (Fig 1B). rCBV_error_ was thus close to 1 for rats in the normal group, which did not have blood vessels with BBB disruption. In rats in the normal group, the AUC ratios were 0.613 and 0.596 for the right and left hemispheres, respectively. The corresponding rCBV_error_ values were 1 (by definition) and 0.971 for the right and left hemispheres, respectively. In rats subjected to tMCAO, the AUC ratios were 0.598 and 0.823 for the contralateral and ipsilateral hemispheres, respectively. This indicates post-ischemic hyper-perfusion caused by the SPION injection in the ipsilateral (ischemic) brain hemisphere [1]. The same hyper-perfusion was not observed following the Dotarem injection, presumably due to CBV and CBF underestimations associated with BBB leakage. The corresponding rCBV_error_ values were 1 (by definition) and 1.376 for the right and left hemispheres, respectively. The rCBV_error_ values for the six rats in the stroke group in this study are summarized in Table 1.

**Table 1:** Mean and standard variations of ADC and rCBV error values for infarction, peri-infarction, and normal areas for six rats shown in Figs 4, 5 and 6.

	Rat_1_			Rat_2_			Rat_3_			Rat_4_			Rat_5_			Rat_6_		
	ADC (×10^6^ μm^2^/s)	rCBV error (a.u.)	rCBV (a.u.)	ADC (×10^6^ μm^2^/s)	rCBV error (a.u.)	rCBV (a.u.)	ADC (×10^6^ μm^2^/s)	rCBV error (a.u.)	rCBV (a.u.)	ADC (×10^6^ μm^2^/s)	rCBV error (a.u.)	rCBV (a.u.)	ADC (×10^6^ μm^2^/s)	rCBV error (a.u.)	rCBV (a.u.)	ADC (×10^6^ μm^2^/s)	rCBV error (a.u.)	rCBV (a.u.)
Normal tissue	715 ± 105	1.063 ± 0.088	1.015 ± 0.270	672 ± 85	1.006 ± 0.109	1.153 ± 0.339	698 ± 86	0.991 ± 0.088	1.070 ± 0.364	681 ± 87	1.023 ± 0.081	1.217 ± 0.524	695 ± 79	0.944 ± 0.096	1.147 ± 0.400	725 ± 85	0.890 ± 0.227	1.138 ± 0.589
Peri-infarct	(*) 645 ± 96	1.321 ± 0.189	1.124 ± 0.365 (^)	(*) 641 ± 77	1.318 ± 0.224	1.388 ± 0.470	674 ± 59	1.274 ± 0.119	1.383 ± 0.468	(*) 618 ± 75	1.254 ± 0.081	1.197 ± 0.542	(*) 642 ± 100	1.295 ± 0.183	1.379 ± 0.592	(*) 672 ± 94	1.481 ± 0.190	1.574 ± 0.593 (^)
Infarct	461 ± 21	1.207 ± 0.283	0.927 ± 0.237	-	-	-	-	-	-	418 ± 38	1.219 ± 0.105	1.315 ± 0.444	413 ± 41	1.248 ± 0.143	1.326 ± 0.420	437 ± 29	1.498 ± 0.147	1.354 ± 0.361

* (p < 0.01) denotes Wilcoxon rank-sum test results for ADC values between normal and peri-infarct zones.

^ (p < 0.01) denotes Wilcoxon rank-sum test results for rCBV values between peri-infarct and infarct zones. a.u. means arbitrary unit.

### Quantified DSC-derived perfusion errors for normal and post-ischemic rat brains

**Fig 2:**
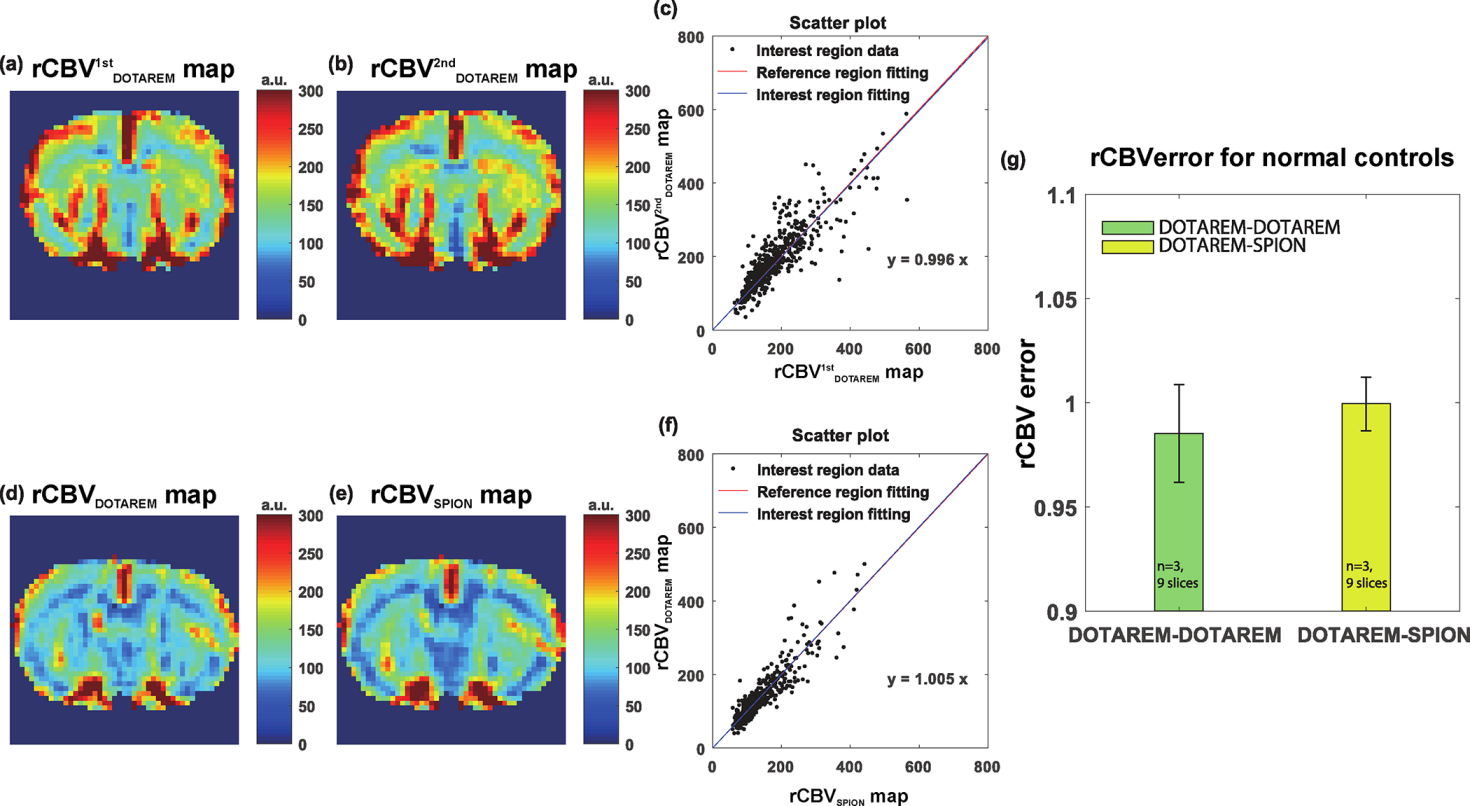
Results of in vivo experiment with normal rats (control experiments). (a) and (b) show the normalized AUC from the first injection (nAUC_Dotarem_) and the AUC from the second injection (AUC_Dotarem_), respectively. (c) Scatter plot of nAUC_Dotarem_ and AUC_Dotarem_ in the left hemisphere (black dots). The red and blue lines are fitted lines to the right- and left-hemisphere scatter plots, respectively. (d) and (e) show the normalized AUC from the first Dotarem injection (nAUC_Dotarem_) and the AUC from the second SPION injection (AUC_SPION_), respectively. (f) Scatter plot of nAUC_Dotarem_ and AUC_SPION_ in the left hemisphere (black dots). The red and blue lines are the corresponding fitted lines to the right- and left-hemisphere scatter plots. (g) Statistical analysis of nAUC ratio. The statistical unit n_sl_ is the number of slices. The green and yellow bar graphs show the nAUC ratio values in the Dotarem-Dotarem and Dotarem-SPION cases, respectively, in normal rats (green bar: 0.990 ± 0.023 [mean ± standard deviation, *n* = 3, with 9 slices], yellow bar: 1.002 ± 0.017 [*n* = 3 with 9 slices], p > 0.05 [p = 0.136]). a.u. means arbitrary unit.

Fig 2 shows the rCBV results for the Dotarem-Dotarem and Dotarem-SPION injections in normal rat brains, respectively. Fig 2A and 2B show the rCBV^1st^_Dotarem_ (first injection) and rCBV^2nd^_Dotarem_ (second injection) maps, which are used as controls, respectively. No noticeable differences were observed between the maps. Fig 2C displays the scatterplot of rCBV^1st^_Dotarem_ values against rCBV^2nd^_Dotarem_ values in the left hemisphere. Fig 2D and 2E show the rCBV_Dotarem_ (first injection) and rCBV_SPION_ (second injection) maps, respectively. No noticeable difference was observed between these maps, either. Finally, Fig 2F presents the scatterplot of rCBV_Dotarem_ values against rCBV_SPION_ values in the left hemisphere. The absence of significant differences (rCBV_error_ values were close to 1 for rats in the normal group) between rCBV^1st^_Dotarem_ and rCBV^2nd^_Dotarem_, and rCBV_Dotarem_ and rCBV_SPION_ confirm the consistency of the rCBV measurements obtained following successive CA injections of Dotarem and SPION (criterion standard) in non-leaking vasculature (normal condition) [22]. Fig 2G summarizes the statistical analysis results for each group. There were no significant differences (Wilcoxon rank sum test, p > 0.05, p = 0.136).

**Fig 3:**
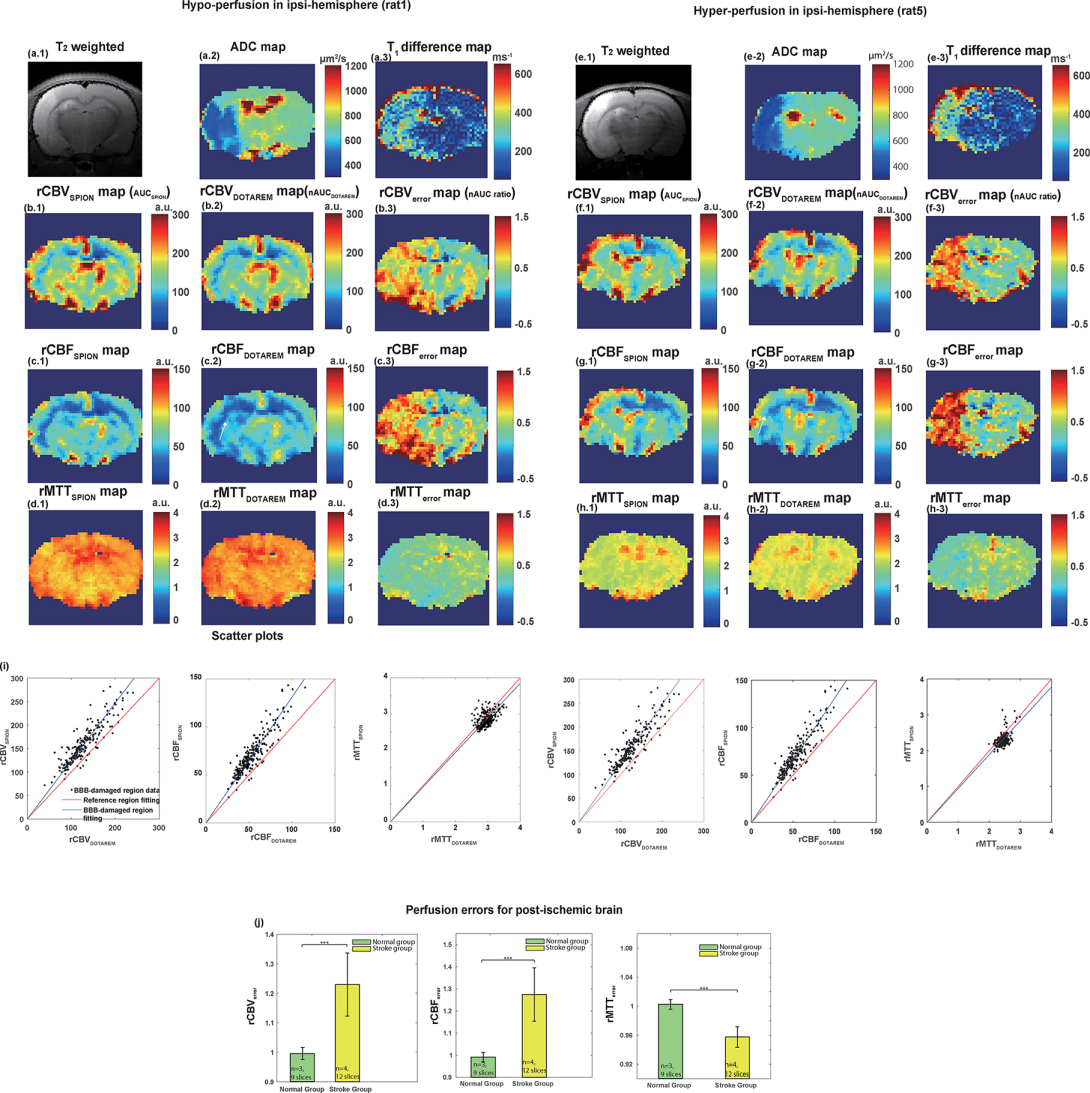
Results of in vivo experiment on two stroke rats with local hypo- and hyper-perfusion. (a.1, e.1) *T*_*2*_-weighted images, (a.2, e.2) ADC maps, and (a.3, e.3) *T*_*1*_ map difference between pre- and post-Dotarem injection. (b.1, f.1) and (b.2, f.2) show the rCBV_SPION_ (AUC_SPION_) and rCBV_Dotarem_ (nAUC_Dotarem_), respectively. (b.3, f.3) show the associated rCBV_error_ (nAUC ratio). (c.1, g.1) and (c.2, g.2) show the rCBF_SPION_ and rCBF_Dotarem_, respectively. (c.3, g.3) show the associated rCBF_error_. (d.1, h.1) and (d.2, h.2) show the rMTT_SPION_ and rMTT_Dotarem_, respectively. (d.3, h.3) show the associated rMTT_error_. (i) Respective scatter plots of rCBV_SPION_/rCBV_Dotarem_, rCBF_SPION_/rCBF_Dotarem_, and rMTT_SPION_/rMTT_Dotarem_ in the damaged BBB ipsi-lesional hemisphere of stroke (black dots). The red and blue lines are fitted lines for the contralateral hemisphere and the BBB-disrupted lesion, respectively. (j) Respective statistical analysis of corresponding perfusion parameters. The green and yellow bar graphs represent the values from the normal (*n* = 3 with 9 slices) and stroke groups (*n* = 4 with 12 slices), respectively. a.u. means arbitrary unit.

Fig 3 shows plots of cerebral perfusion measurement errors, such as rCBV_error_, rCBF_error,_ and rMTT_error_, for cases of post-ischemic local hypo- and hyper-perfusion. As seen in the ADC maps (Fig 3A2 and 3E2), the decreased ADC values and hyperintense *T*_*2*_-weighted images (Fig 3A1 and 3E1) of the left hemisphere indicate the presence of ischemic stroke lesions after tMCAO reperfusion. Fig 3A3 and 3E3 illustrate the differences in the *T*_1_ maps before and after the Dotarem injection. The significant differences in *T*_1_ values in the ipsilateral hemisphere provide a clear indication of Dotarem leakage. No differences in *T*_1_ values were observed following the SPION injections. Fig 3B1 and 3F1 and Fig 3B2 and 3F2, show the rCBV_SPION_ and the rCBV_Dotarem_ maps, respectively, while Fig 3B3 and 3F3 show the resulting rCBV_error_ due to CA extravasation. Fig 3C1 and 3G1, and Fig 3C2 and 3G2, show the rCBF_SPION_ and the rCBF_Dotarem_ maps, respectively, while Fig 3C3 and 3G3 show the resulting rCBF_error_. Fig 3D1 and 3H1 and Fig 3D2 and 3H2, show the rMTT_SPION_ and rMTT_Dotarem_ maps, respectively, while Fig 3D3 and 3H3 show the resulting rMTT_error_. In the case of hypo-perfusion, both rCBF_SPION_ and rCBF_Dotarem_ indicate the presence of post-ischemic ipsilateral hypo-perfusion with respect to the contralateral hemisphere, as indicated by the white arrow in Fig 3C2. In contrast, in the case of hyper-perfusion, rCBF_SPION_ indicates the presence of significant post-ischemic hyper-perfusion, while rCBF_Dotarem_ has non-significant differences with respect to the contralateral hemisphere, as indicated by the white arrow in Fig 3G2. These results clearly indicate that the DSC-MRI-derived CBV and CBF values were significantly underestimated when using gadolinium chelates, and in this case, led to errors in the detection of post-ischemic hyper-perfusion. In the ipsi-stroke hemisphere in both cases, the corresponding rCBV_error_ and rCBF_error_ were apparent not only in the infarcted zone (ADC < 500 μm^2^/s), but also in the peri-infarcted area. The boundaries of the region with significant rCBV_error_ and rCBF_error_ values mostly matched those of the corresponding *T*_1_ difference map, indicating the leakage of Dotarem. The BBB leakage-derived errors did not appear to correlate with the *V*_*p*_ (rCBV_SPION_) values. It was also noted that when compared to the values obtained following the SPION injection, the rCBV_Dotarem_ and rCBF_Dotarem_ values were generally underestimated, and the rMTT_Dotarem_ value was slightly overestimated. Fig 3I shows differences in the slopes of the scatterplots for the three perfusion indices obtained following the use of Dotarem and SPION in the BBB-disrupted region. The ROIs for these scatterplots were obtained from the corresponding *T*_1_ difference maps. Rats in the normal group were used as controls. The perfusion parameters calculated in these rats were compared to those calculated in rats in the stroke group (Fig 3J). The rCBV_error_ values for lesions in BBB-disrupted regions differed significantly from those obtained in rats in the control group (Wilcoxon rank sum test, p < 0.01, p = 1.98 × 10^−7^). In other words, the rCBV_Dotarem_ values for lesions in the BBB-disrupted regions were estimated to be significantly lower (~20%) than the corresponding rCBV_SPION_ values.

### Regional segmentation of post-ischemic brain based on rCBV_error_, ADC, and *V*_*p*_

**Fig 4:**
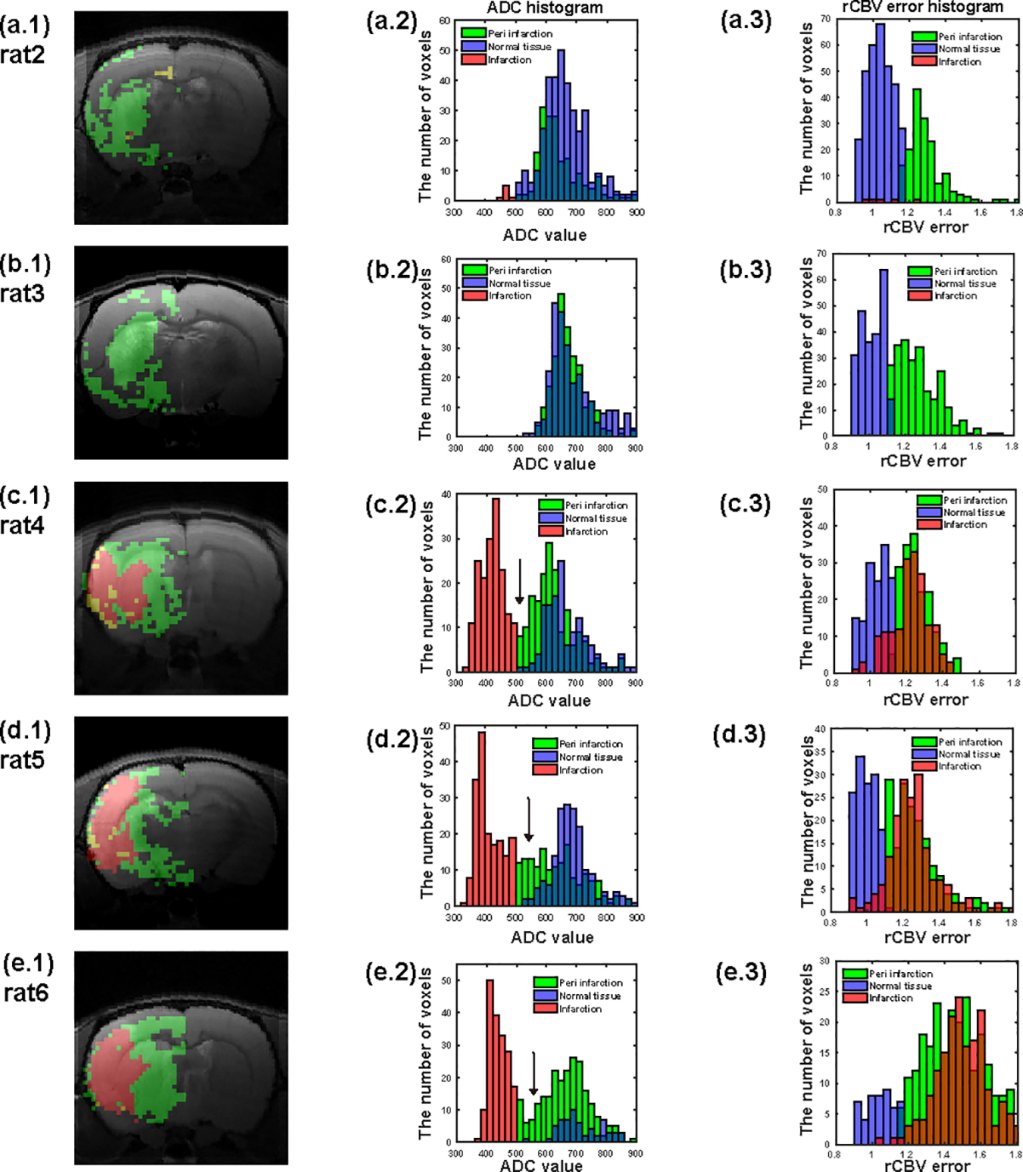
(a.1), (b.1), (c.1), (d.1), (e.1) show infarcted areas with BBB damage (ADC < 500 μm^2^/s and rCBV_error_ > threshold) in red and peri-infracted areas with BBB damage (ADC > 500 μm^2^/s and rCBV_error_ > threshold) in green. Infarcted area without BBB damage (ADC < 500 μm^2^/s and rCBV_error_ < threshold) is shown in yellow, but scarce. (a.2), (b.2), (c.2), (d.2), (e.2) show histograms of ADC values of corresponding infarcted (red), per-infarcted with BBB-damage (green), and normal (purple) areas. The normal region satisfies the condition of ADC > 500 μm^2^/s and rCBV_error_ < threshold. The overlapping ADC values between peri-infarcted (green) and normal (purple) areas are shown in dark blue. (a.3), (b.3), (c.3), (d.3), (e.3) show histograms of rCBV_error_ values corresponding to infarcted (red), per-infarcted with BBB damage (green), and normal (purple) areas. The overlapping rCBV_error_ values between infarcted (red) and peri-infarcted (green) areas are shown in dark orange.

In the first column of Fig 4, the infarct (ADC < 500 μm^2^/s) voxels of regions with BBB damage (rCBV_error_ > threshold) are shown in red. The infarct (ADC < 500 μm^2^/s) voxels of regions without BBB damage (rCBV_error_ < threshold) are shown in yellow, but are scarce. Voxels of BBB-damaged regions (rCBV_error_ > threshold) without infarction (ADC > 500 μm^2^/s) are shown in green. The green area may thus represent the peri-infarct area where there is BBB damage. In the second column of Fig 4, the ADC histograms for the infarct (ADC < 500 μm^2^/s), peri-infarct with BBB damage (ADC > 500 μm^2^/s and rCBV_error_ > threshold), and normal (ADC > 500 μm^2^/s and rCBV_error_ < threshold) regions are shown in red, green, and purple, respectively. The overlapping ADC values between the peri-infarct (green) and normal (purple) areas are shown in dark blue. For the small lesions shown in the top two rows, the peri-infarct areas had noticeably smaller ADC values than areas of normal tissue. For the larger lesions shown in the bottom three rows, the presence of non-overlapping mid-range ADC values (500 μm^2^/s < ADC < 600 μm^2^/s) was distinct in the peri-infarct areas when compared to areas of normal tissue (indicated by black arrows on the ADC histograms). In the third column of Fig 4, rCBV_error_ histograms for the infarct, peri-infarct with BBB damage, and normal regions are shown in red, green, and purple, respectively. The overlapping rCBV_error_ values between the infarct (red) and peri-infarct (green) areas are shown in dark orange. No significant differences were observed in the rCBV_error_ values between the infarct (red) and peri-infarct (green) areas, as most of the red-tagged values overlap with green-tagged areas.

Table 1 summarizes the ADC, rCBV_error_, and rCBV_SPION_ (*V*_*p*_) values for each region for the six rats subjected to stroke. The ADC values were significantly different for the infarct, peri-infarct, and normal regions. The rCBV_error_ values were significantly different between the infarct (peri-infarct) and normal regions, but no difference was observed between the infarct and peri-infarct regions. The rCBV_SPION_ (*V*_*p*_) values did not appear to strongly correlate with the applied regional segmentation.

### Leakage compensation with CA pre-load

**Fig 5:**
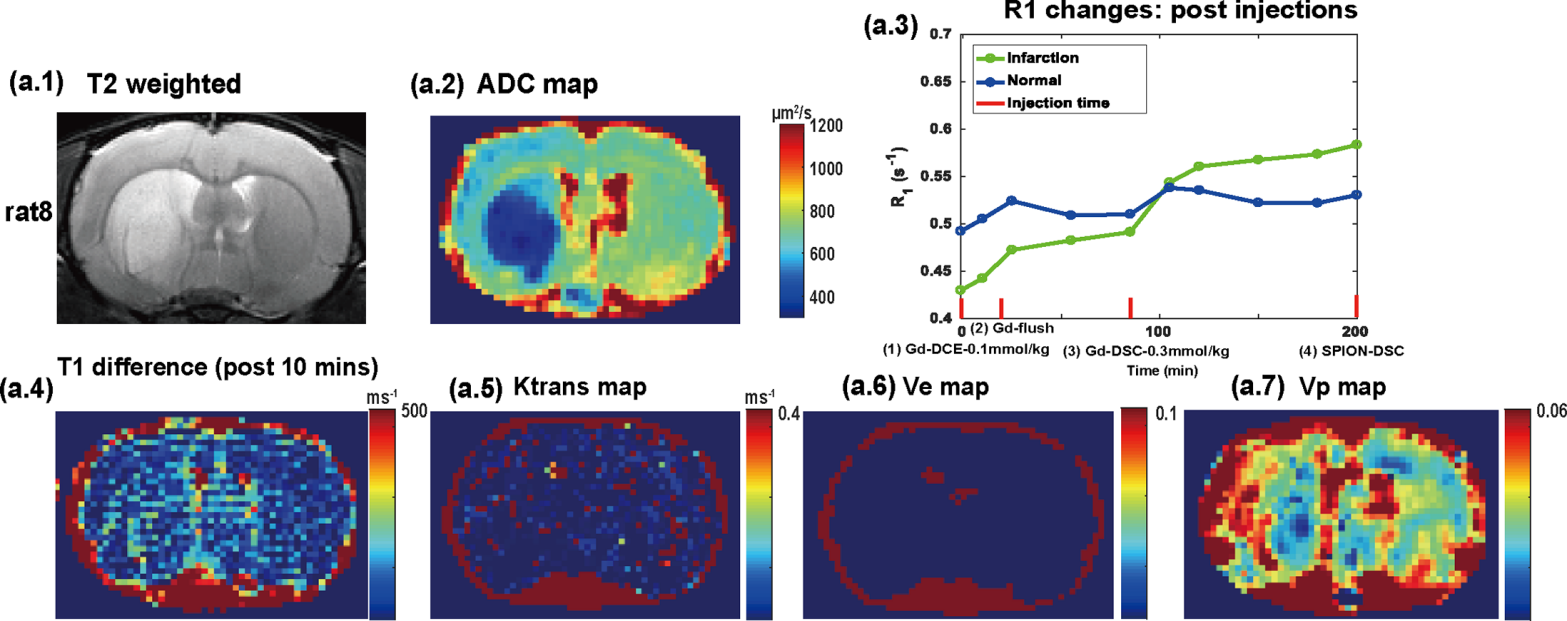
(a.1) *T*_*2*_-weighted image. (a.2) ADC map. (a.3) *T*_1_ changes were shown for infarction (green) and normal (blue) region as a function of post-injection time, where injection time-points were marked with red lines. No significant *T*_1_ difference between infarction and normal region were observed from (a.4) *T*_1_ difference map (post-10 mins) after the injection of DOTAREM (0.1 mmol·kg^-1^). (a.5) *K*^*trans*^ map. (a.6) *V*_*e*_ map. (a.7) *V*_*p*_ map.

We performed DCE-MRI acquisitions before the DSC-MRI measurements with both Dotarem and SPION in three post-ischemic rats (a *T*_*2*_-weighted image and an ADC map for a representative animal are shown in Fig 5A1 and 5A2, respectively). *R*_*1*_ (= *1/T*_*1*_) changes are shown for infarcted (green) and normal (blue) regions as a function of post-injection time, where the injection time-points are marked with red lines on the axis in Fig 5A3. No significant *T*_1_ differences (after 10 min) were observed pre- vs. post-injection of Dotarem (0.1 mmol·kg^-1^) in the infarction and normal regions, as shown in Fig 5A4. Similarly, no significant differences in vessel wall permeability (*K*^*trans*^) values obtained using the extended Toft model were observed in the infarction region, as shown in Fig 5A5. After the Dotarem flush (0.2 mmol·kg^-1^ after 15 min), the *T*_1_ values were further reduced in the infarction region, but not in the normal region. This led to a significant difference in *T*_1_ (after 85 min) pre- vs. post-injection, as shown in Fig 5A3.

**Fig 6:**
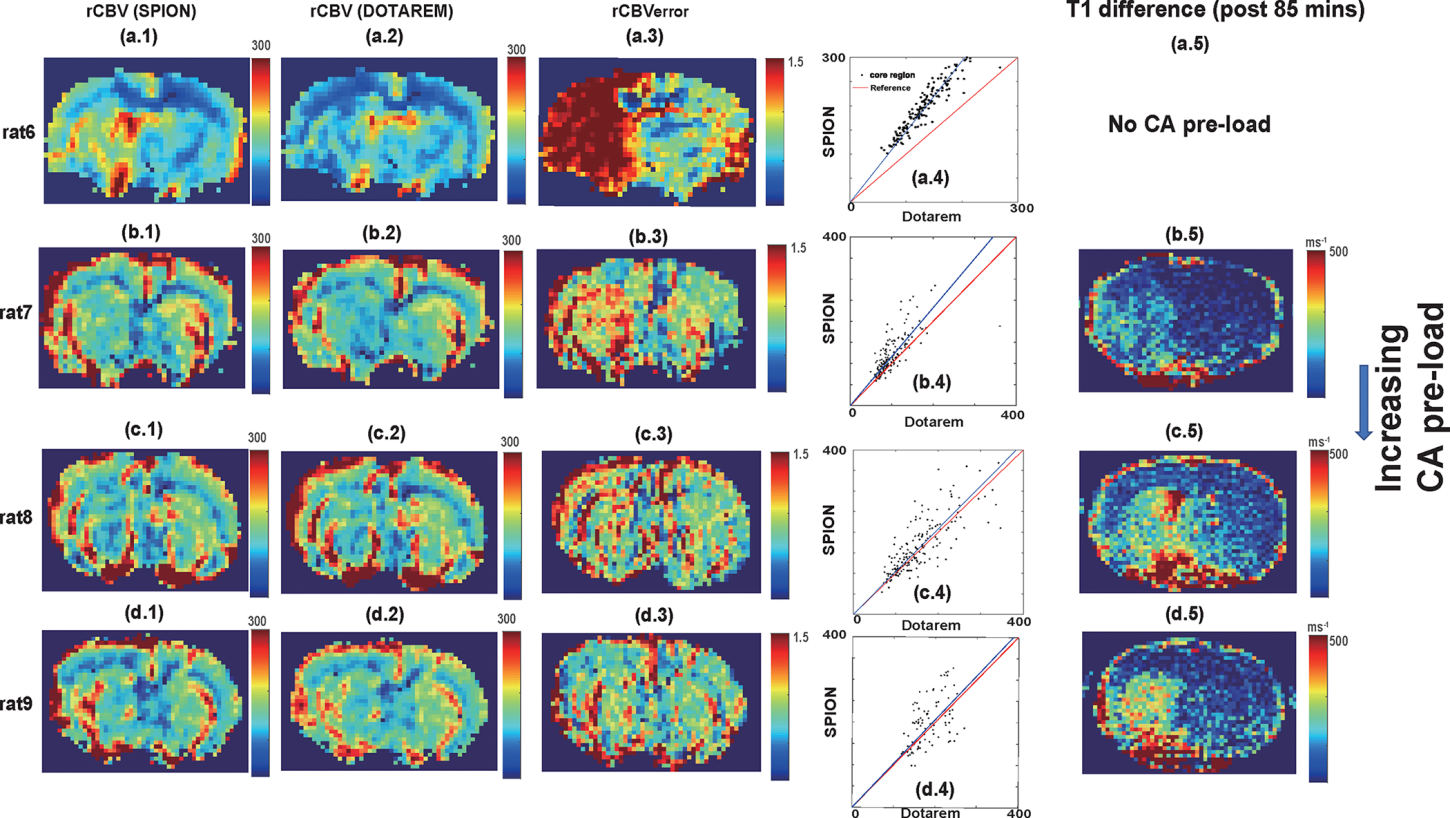
(a.1-d.1) rCBV maps from SPION. (a.2-d.2) rCBV maps from DOTAREM (a.3-d.3) rCBV_error_ map. (a.4-d.4) Scatter plots of rCBV_SPION_/rCBV_DOTAREM_ values in core region normalized to each normal area. (a.5-d.5) *T*_*1*_ difference maps (post 85 mins) with increasing CA pre-load. No *T*_*1*_ difference map was available for no CA pre-load case and not available.

Perfusion deficits identified using fast DSC-MRI following increasing CA pre-loads are shown in Fig 6. Significant underestimation of rCBV_Dotarem_ with respect to rCBV_SPION_ without CA pre-load was again apparent, as shown in Fig 6A1, 6A2 and 6A3. However, in the presence of significant CA pre-load (net: 0.3 mmol·kg^-1^), the SPION and Dotarem rCBV maps were similar and there was minimal rCBV_error,_ as shown in Fig 6C1, 6C2 and 6C3. In another rat with a comparable *T*_*1*_ difference map and CA pre-load (net: 0.3 mmol·kg^-1^), we repeatedly observed significantly reduced rCBV_error_, as shown in Fig 6D1, 6D2 and 6D3. In contrast, in a rat with a less conspicuous *T*_*1*_ difference map (Fig 6B5), a noticeable underestimation of rCBV_Dotarem_ was still observed, as shown in Fig 6B1, 6B2 and 6B3. Fig 6A4, 6B4, 6C4 and 6D4 compare scatterplots of rCBV values for the core region following Dotarem and SPION injections. Mismatches between the core and normal areas are reduced with increasing CA pre-load, as shown in the enlargement of the *T*_*1*_ difference map (after 85 min, just before the DSC-MRI acquisition) shown in Fig 6A5, 6B5, 6C5 and 6D5. No significant *T*_*1*_ changes were observed in the rat without CA pre-load (Fig 6A5, corresponding *T*_1_ differences map is not shown).

## Discussion and conclusions

Significant perfusion deficits (rCBV_error_ ~20%) were present in DSC-MRI data obtained using fast (0.3 s) EPI acquisition, even in brains of post-ischemic rats (1-h MCA0, 24-h reperfusion) with insignificant permeability (*K*^*trans*^) values obtained using DCE-MRI with gadolinium chelates administered at the conventional dose of 0.1 mmol·kg^-1^. Even though it is difficult to separate the effects of *T*_*1*_ shortening and susceptibility contrast changes from those of leaking CA on DSC-MRI-derived perfusion deficits, fast EPI acquisition will be inevitably affected by *T*_*1*_ shortening due to the leaky vessels. Nevertheless, in animal model studies with rapid blood circulation at high magnetic fields, the lengthening of temporal resolution or the increasing of injection dose may not be adequate for the accurate determination of perfusion parameters. As a result, leakage compensation using CA pre-load (0.3 mmol·kg^-1^) may be necessary to avoid perfusion deficits during high temporal resolution DSC-MRI acquisition in animal models of early post-ischemic reperfusion. This is especially true for longitudinal follow-up studies of post-ischemic animal models with varying degrees of vessel wall permeability [14].

It is also worthwhile to note that the DSC signal bias originates from compromised BBB integrity and is likely to be proportional to *K*^*trans*^ and inversely proportional to CBV. As a result, the perfusion errors in DSC-MRI data obtained using extravasating vs. intravascular CAs may be particularly sensitive to weakly leaking microvessels with small CBVs. The conspicuous rCBV_error_ in this tMCAO model may thus provide more sensitive diagnostic information than the corresponding ADC and rCBV_SPION_ (*V*_*p*_) maps. The increased area of the elevated rCBV_error_ values for enlarging infarct regions may articulate the boundaries of the BBB-disrupted areas in the ipsilesional hemisphere in this ischemic reperfusion stroke model. The significantly larger regions with disrupted BBB than the diffusion-reduced (infarction) regions in the tMCAO model indicate peri-infarct BBB-damaged capillaries (Fig 3), which may lead to secondary vascular dysfunction that can limit recovery of viable tissue near an infarcted zone. A few histology-based studies have also confirmed the existence of BBB-damaged capillaries in peri-infarct zones [23,24]. In the peri-infarct BBB-damaged zone determined based on the rCBV_error_ threshold, a significant reduction in the ADC value was apparent between 500 μm^2^/s and 600 μm^2^/s (Fig 3 and Table 1). The region with slightly decreased ADC values co-localized with the elevated rCBV_error_, and is likely to be a signature of early vasogenic edema, some of which may progress to irreversible infarction.

The potential limitations of this study are as follows. First, direct histological comparison was not possible for the proposed rCBV_error_ values, as independent three-dimensional characterization methods for vessel permeability and flow are scarce. Instead, considering the similar molecular weight of Dotarem (0.56 kDa) to that of traditional Evans blue dye (0.9 kDa), the *T*_*1*_ difference maps obtained before and after the Dotarem injection (0.3 mmol·kg^-1^) were used to assess BBB leakage in this study. Second, any Dotarem remaining in the tissue may impose a signal bias for the following DSC-MRI performed after the SPION injection. However, as the Δ*R*_2_*(t) of the early-phase DSC-MRI was obtained based on the signal differences before vs. after the CA administration rather than from the absolute signals, we assumed that the effects of remaining CA on the second injection were minimal. Future developments in fast *T*_1_ acquisition methods with improved sensitivity [25,26] or the complete separation of *T*_2_* and *T*_1_ changes in the first-passage signal time courses for the leaky BBB may provide further insights into the biophysical mechanisms underlying multiple CA injections. Third, although the Δ*R*_2_*(t) values obtained at 7 T are minimally related to the underlying vessel sizes or shapes [27] and are primarily dependent on CBV and CBF values for randomly oriented vessels, cautious interpretation of *T*_2_^*^-based DSC-MRI signals is required due to potential geometric complications and unwanted susceptibility artifacts in post-ischemic brain applications. Future investigation of the effects of unaccounted Dotarem-cell interactions or vessel size effects on dual DSC-MRIs may be pursued in post-ischemic animal models of weak BBB damage.

In summary, we combined two different AUC measurements of sequential DSC-MRIs to characterize cerebral perfusion errors using extravasating (Dotarem) and intravascular (SPION) CAs in the brains of a post-ischemic 60-minute MCAO and 1-day reperfusion rat model. DSC-MRI-derived perfusion indices, such as relative CBF and CBV values obtained using an extravasating CA, were underestimated (~20%). The brain area with a significant rCBV_error_ encompassed the region of infarct tissue and mostly co-localized with the region with *T*_*1*_ differences pre- vs. post-Dotarem injection. This indicates the presence of a disrupted BBB in the infarct and peri-infarct regions. The DSC measurements obtained using significant pre-load (0.3 mmol·kg^-1^) of Dotarem had minimal perfusion deficits when compared to those obtained using the reference intravascular SPION.

## Supporting information

S1 Fig (a-1) Dynamic *ΔR*_2_^*^ curve from SPION in normal rat brain. (a-2) The ROI of the brain location, where the *ΔR*_2_^*^ curve (a-1) is sampled from. Time intervals of first passage (pre-injection~4s), second passage (4~10s) and steady state (>10s) were defined based on DSC-MR signal with intravascular SPION injection. (b) The ipsilateral (red) and contralateral (green) ROI of the brain locations, where the *ΔR*_2_^*^ curves of stroke rats were sampled for Fig 1C.
(TIF)

S2 Fig Experimental schemes of animal preparation and MR scan. (a) Experimental scheme for normal rats. (b) Experimental scheme for stroke group rats. A *T*_*2*_-weighted image, ADC map, and *T*_*1*_ map were additionally obtained for the stroke group rats. The durations for *T*_*2*_-weighted image (RARE), ADC map (DW-EPI), *T*_*1*_ map (RAREVTR), and AUC map (DSC-EPI) acquisitions were 10, 9, 10, and 4 mins, respectively. The duration between the injections for each experiment was 2 hours. The duration for MCAO and following reperfusion was 1 hour and 24 hours, respectively.
(TIF)

S3 Fig The process of nAUC estimation. (a) and (b) AUC_DOTAREM_ and AUC_SPION_ map from normal rat brain. (c) Scatter plot between AUC_DOTAREM_ and AUC_SPION_ for reference and interest region. (d) nAUC_DOTAREM_ map, which was divided by the ratio of AUC_SPION_ and AUC_DOTAREM_ (= 0.573). (e) Scatter plot between nAUC_DOTAREM_ and AUC_SPION_ for reference and interest region.
(TIF)

S4 Fig (a) The *T*_*1*_ difference before and after the CA injection for 1-hr MCAO and 1-day reperfusion model. The green and yellow bar graphs present the *T*_*1*_ difference of the ipsilateral infarction (ADC < 500 μm^2^/s) and contralateral regions, respectively. The statistical unit n_sl_ is the number of slices. For the DOTAREM case (left), green bar: 252 ± 94 ms (n_sl_ = 12), yellow bar: 87 ± 39 ms (n_sl_ = 12), and p < 0.01 (p = 0.001). For the SPION case (right), green bar: 27 ± 31 ms (n_sl_ = 12), yellow bar: 47 ± 26 ms (n_sl_ = 12), and p > 0.05 (p = 0.229). (b) The *T*_*1*_ difference maps for 1-hr MCAO/1-day and 1-hr MCAO/7-day reperfusion models, respectively. Significant leakage of SPION is apparent in 1-hr MCAO/7-day reperfusion model.
(TIF)

S5 Fig Respective rCBV histograms of normal, peri-infarction, and infarction regions from six stroke rats, which were reported in Table 1. (TIF)

S6 Fig The fittings of DCE-MRI time curves, which were used to generate *K*_*trans*_, *V*_*e*_, and *V*_*p*_ maps shown in Fig 5. Blue and red dots represent time-signal data for normal and infarction regions, respectively.
(TIF)

S7 Fig The fittings of ADC values, which were used to generate ADC maps shown in Figs 3–5. Blue and red dots represent diffusion data for normal and infarction regions, respectively for six stroke rats.
(TIF)

S8 Fig The fittings of rCBV values from DSC-MRI, which were used to generate rCBV maps throughout the manuscript. (TIF)

## References

[1] Garcia JH, Experimental ischemic stroke: A review. Stoke 1984;15: 5–14. 10.1161/01.str.15.1.5 6364464

[2] Hossmann K-A, Animal models of cerebral ischemia: I. Review of literature. Cerebrovascular Diseases. 1991;1: 2–15.

[3] Shen Q, Du F, Huang S, Duong TQ. Spatiotemporal characteristics of post-ischemic hyper-perfusion with respect to changes in T1, T2, diffusion, angiography, and blood–brain barrier permeability. Journal of Cerebral Blood Flow & Metabolism. 2011;31(10): 2076–2085. 21540871 10.1038/jcbfm.2011.64 PMC3208152

[4] Shen Q, Duong TQ. Magnetic resonance imaging of cerebral blood flow in animal stroke models. Brain Circulation. 2016;2(1): 20 10.4103/2394-8108.178544 26998527 PMC4797655

[5] Calamante F, Thomas DL, Pell GS, Wiersma J, Turner R. Measuring cerebral blood flow using magnetic resonance imaging techniques. Journal of Cerebral Blood Flow & Metabolism. 1999;19(7): 701–735. 10413026 10.1097/00004647-199907000-00001

[6] Cha S, Knopp EA, Johnson G, Wetzel SG, Litt AW, Zagzag D. Intracranial Mass Lesions: Dynamic Contrast-enhanced Susceptibility-weighted Echo-planar Perfusion MR Imaging 1. Radiology. 2002;223(1): 11–29. 10.1148/radiol.2231010594 11930044

[7] Zierler KL. Theoretical basis of indicator-dilution methods for measuring flow and volume. Circulation Research. 1962;10(3): 393–407.

[8] Uematsu H, Maeda M. Double-echo perfusion-weighted MR imaging: basic concepts and application in brain tumors for the assessment of tumor blood-volume and vascular permeability. European Radiology. 2006;16(1): 180–186. 10.1007/s00330-005-2807-9 16402258

[9] Boxerman JL, Schmainda KM, Weisskoff RM. Relative cerebral blood-volume maps corrected for contrast agent extravasation significantly correlate with glioma tumor grade, whereas uncorrected maps do not. American Journal of Neuroradiology. 2006; 27(4): 859–867. 16611779 PMC8134002

[10] Vonken EjPA, van Osch MJP, Bakker CJG, Viergever MA. Simultaneous quantitative cerebral perfusion and Gd‐DTPA extravasation measurement with dual‐echo dynamic-susceptibility-contrast MRI. Magnetic Resonance in Medicine. 2000;43(6): 820–827. 10861876 10.1002/1522-2594(200006)43:6<820::aid-mrm7>3.0.co;2-f

[11] Dósa E, Guillaume DJ, Haluska M, Lacy CA, Hamilton BE, Njus JM, et al Magnetic resonance imaging of intracranial tumors: intra-patient comparison of gadoteridol and ferumoxytol. Neuro-oncology. 2010;13(2): 251–60. 10.1093/neuonc/noq172 21163809 PMC3064624

[12] Quarles CC, Ward BD, Schmainda KM. Improving the reliability of obtaining tumor hemodynamic parameters in the presence of contrast agent extravasation, Magnetic Resonance in Medicine. 2005;53(6): 1307–1316. 10.1002/mrm.20497 15906288

[13] Boxerman JL, Prah DE, Paulson ES, Machan JT, Bedekar D, Schmainda KM, The role of preload and leakage correction in Gadolinium-based cerebral blood volume estimation determined by comparison with MION as a criterion standard, American Journal of Neuroradiology, 2012;33(6): 1081–1087 10.3174/ajnr.A2934 22322605 PMC4331024

[14] Lin CY, Chang C, Cheung WM, Lin MH, Chen JJ, Hsu CY, et al Dynamic changes in vascular permeability, cerebral blood volume, vascular density, and size after transient focal cerebral ischemia in rats: evaluation with contrast-enhanced magnetic resonance imaging. Journal of Cerebral Blood Flow & Metabolism. 2008;28(8): 1491–501. 18478021 10.1038/jcbfm.2008.42

[15] Tanaka Y, Nagaoka T, Nair G, Ohno K, Duong TQ. Arterial spin labeling and dynamic susceptibility contrast CBF MRI in postischemic hyperperfusion, hypercapnia, and after mannitol injection. Journal of Cerebral Blood Flow & Metabolism. 2011;31(6): 1403–1441. 21179070 10.1038/jcbfm.2010.228 PMC3130313

[16] Lee DK, Han SH, Cho H. Optimization of Sparse Phase Encodings for Variable-Repetition-Delay Turbo-Spin Echo (TSE) T1 Measurements for preclinical applications, Journal of Magnetic Resonance, 2017;274: 57–64. 10.1016/j.jmr.2016.11.004 27886558

[17] Hennig J, Nauerth A, Friedburg H. RARE imaging: a fast imaging method for clinical MR, 1986, Magnetic Resonance in Medicine, 1986;3: 823–833. 3821461 10.1002/mrm.1910030602

[18] Heisen M, Fan X, Buurman J, van Riel NA, Karczmar GS, ter Haar Romeny BM. The influence of temporal resolution in determining pharmacokinetic parameters from DCE‐MRI data. Magnetic Resonance in Medicine, 2010;63(3): 811–816 10.1002/mrm.22171 20187187 PMC3076555

[19] Gill R, Sibson NR, Hatfield RH, Burdett NG, Carpenter TA, Hall LD, et al A comparison of the early development of ischemic damage following permanent middle cerebral artery occlusion in rats as assessed using magnetic resonance imaging and histology. Journal of Cerebral Blood Flow & Metabolism, 1995;15(1): 1–11. 7798326 10.1038/jcbfm.1995.1

[20] Roberts C, Issa B, Stone A, Jackson A, Waterton JC, Parker GJ. Comparative study into the robustness of compartmental modeling and model‐free analysis in DCE‐MRI studies. Journal of Magnetic Resonance Imaging, 2006; 23(4): 554–563. 10.1002/jmri.20529 16506143

[21] Keston P, Murray A, Jackson A. Cerebral perfusion imaging using contrast-enhanced MRI. Clinical Radiology. 2003;58(7): 505–13. 12834633 10.1016/s0009-9260(03)00130-2

[22] Simonsen CZ, Østergaard L, Vestergaard‐Poulsen P, Røhl L, Bjørnerud A, Gyldensted C. CBF and CBV measurements by USPIO bolus tracking: reproducibility and comparison with Gd‐based values. Journal of Magnetic Resonance Imaging.1999;9(2): 342–347. 10077035 10.1002/(sici)1522-2586(199902)9:2<342::aid-jmri29>3.0.co;2-b

[23] Nahirney PC, Reeson P, Brown CE. Ultrastructural analysis of blood–brain barrier breakdown in the peri-infarct zone in young adult and aged mice. Journal of Cerebral Blood Flow & Metabolism. 2016;36(2): 413–425. 26661190 10.1177/0271678X15608396 PMC4759675

[24] Reeson P, Tennant KA, Gerrow K, Wang J, Weiser Novak S, Thompson K, et al Delayed Inhibition of VEGF Signaling after Stroke Attenuates Blood–Brain Barrier Breakdown and Improves Functional Recovery in a Comorbidity-Dependent Manner. The Journal of Neuroscience. 2015;35(13): 5128–5143. 10.1523/JNEUROSCI.2810-14.2015 25834040 PMC6705411

[25] Bauman G, Johnson KM, Bell LC, Velikina JV, Samsonov AA, Nagle SK, et al Three‐dimensional pulmonary perfusion MRI with radial ultrashort echo time and spatial–temporal constrained reconstruction. Magnetic Resonance in Medicine. 2015;73(2): 555–64. 10.1002/mrm.25158 24604452 PMC4156934

[26] Johnson KM, Fain SB, Schiebler ML, Nagle S. Optimized 3D ultrashort echo time pulmonary MRI. Magnetic Resonance in Medicine. 2013;70(5): 1241–1250. 10.1002/mrm.24570 23213020 PMC4199575

[27] Han SH, Cho JH, Jung HS, Suh JY, Kim JK, Kim YR, et al Robust MR assessment of cerebral blood volume and mean vessel size using SPION-enhanced ultrashort echo acquisition. NeuroImage. 2015;112: 382–9. 10.1016/j.neuroimage.2015.03.042 25818683

