# Supplementary figures and images for: Cerebral blood perfusion deficits using dynamic susceptibility contrast MRI with gadolinium chelates in rats with post-ischemic reperfusion without significant dynamic contrast-enhanced MRI-derived vessel permeabilities: A cautionary note
Source: PLoS One. 2018 Jul 25;13(7):e0201076. doi: 10.1371/journal.pone.0201076 (PMC6059480; doi:10.1371/journal.pone.0201076)

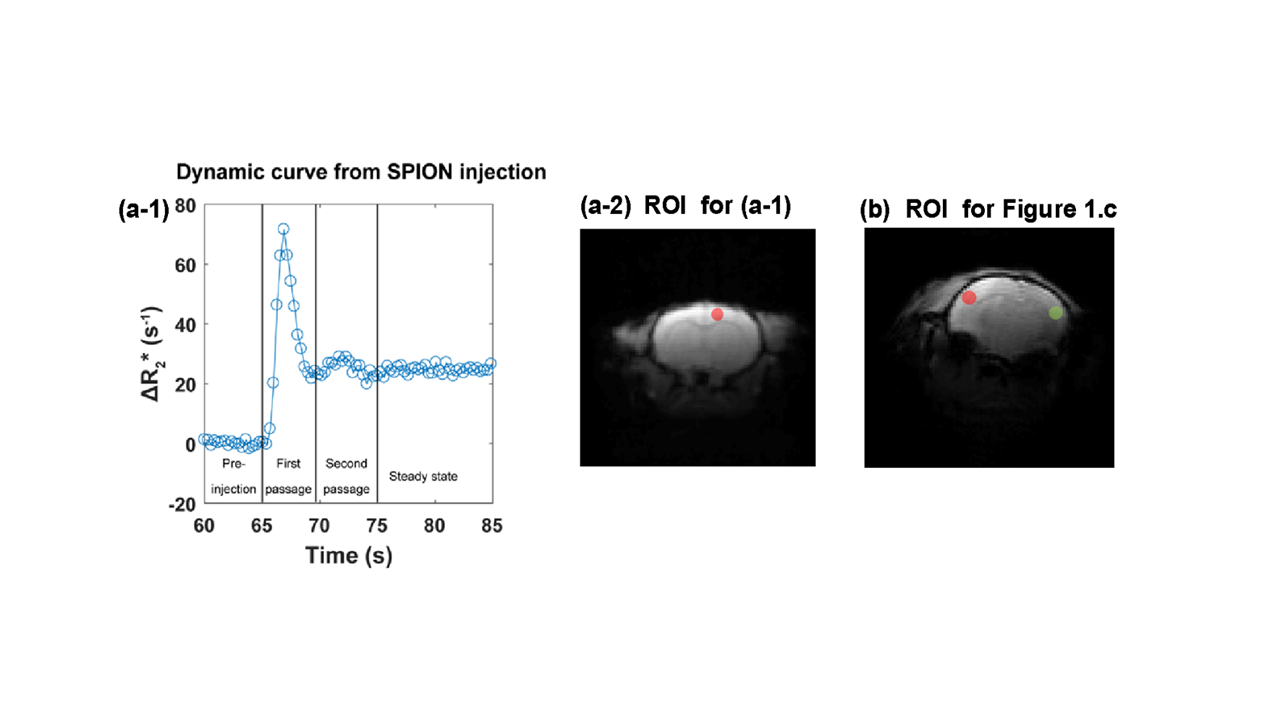

Supplement: S1 Fig — (a-1) Dynamic ΔR2* curve from SPION in normal rat brain. (a-2) The ROI of the brain location, where the ΔR2* curve (a-1) is sampled from. Time intervals of first passage (pre-injection~4s), second passage (4~10s) and steady state (>10s) were defined based on DSC-MR signal with intravascular SPION injection. (b) The ipsilateral (red) and contralateral (green) ROI of the brain locations, where the ΔR2* curves of stroke rats were sampled for Fig 1C. (TIF) [file pone.0201076.s001.tif]

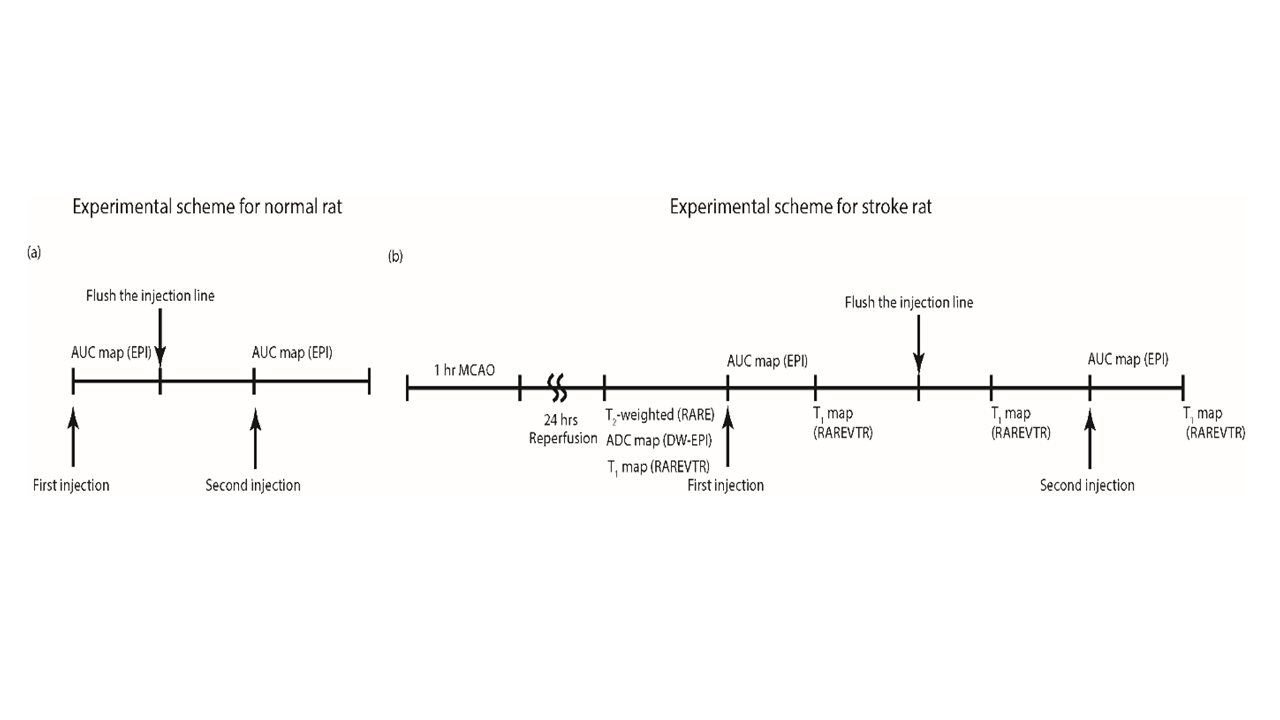

Supplement: S2 Fig — (a) Experimental scheme for normal rats. (b) Experimental scheme for stroke group rats. A T2-weighted image, ADC map, and T1 map were additionally obtained for the stroke group rats. The durations for T2-weighted image (RARE), ADC map (DW-EPI), T1 map (RAREVTR), and AUC map (DSC-EPI) acquisitions were 10, 9, 10, and 4 mins, respectively. The duration between the injections for each experiment was 2 hours. The duration for MCAO and following reperfusion was 1 hour and 24 hours, respectively. (TIF) [file pone.0201076.s002.tif]

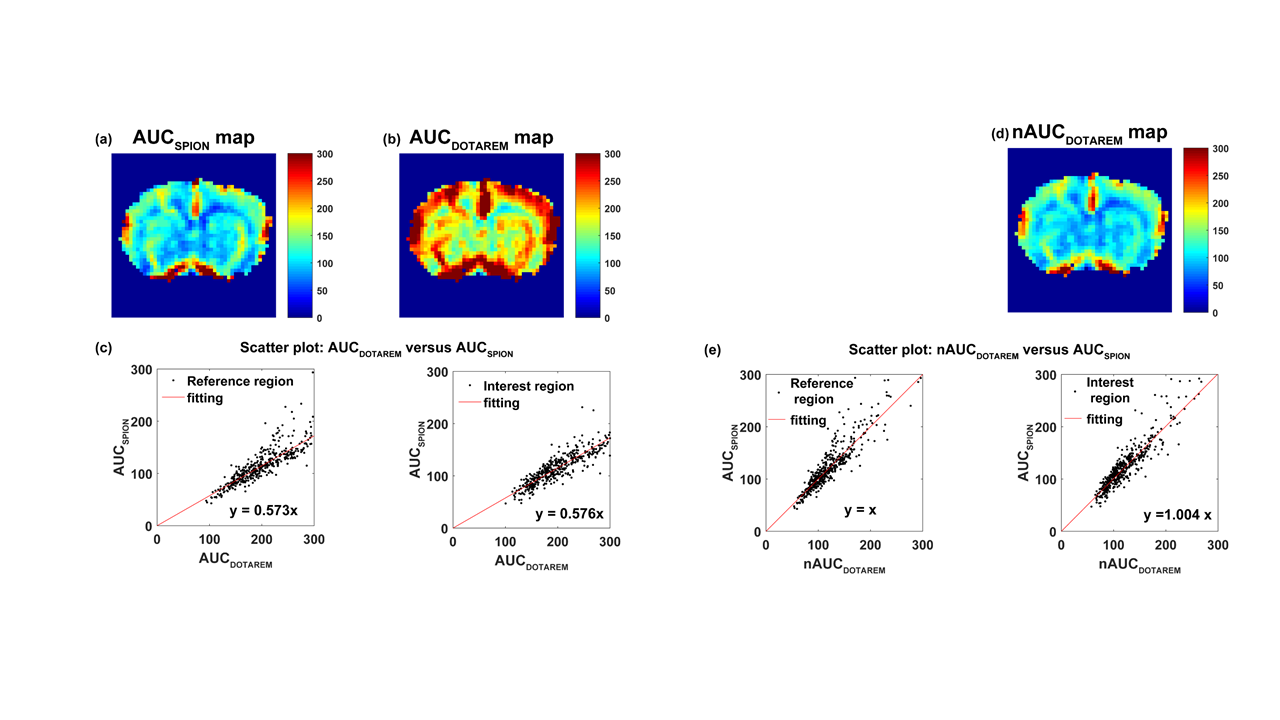

Supplement: S3 Fig — (a) and (b) AUCDOTAREM and AUCSPION map from normal rat brain. (c) Scatter plot between AUCDOTAREM and AUCSPION for reference and interest region. (d) nAUCDOTAREM map, which was divided by the ratio of AUCSPION and AUCDOTAREM (= 0.573). (e) Scatter plot between nAUCDOTAREM and AUCSPION for reference and interest region. (TIF) [file pone.0201076.s003.tif]

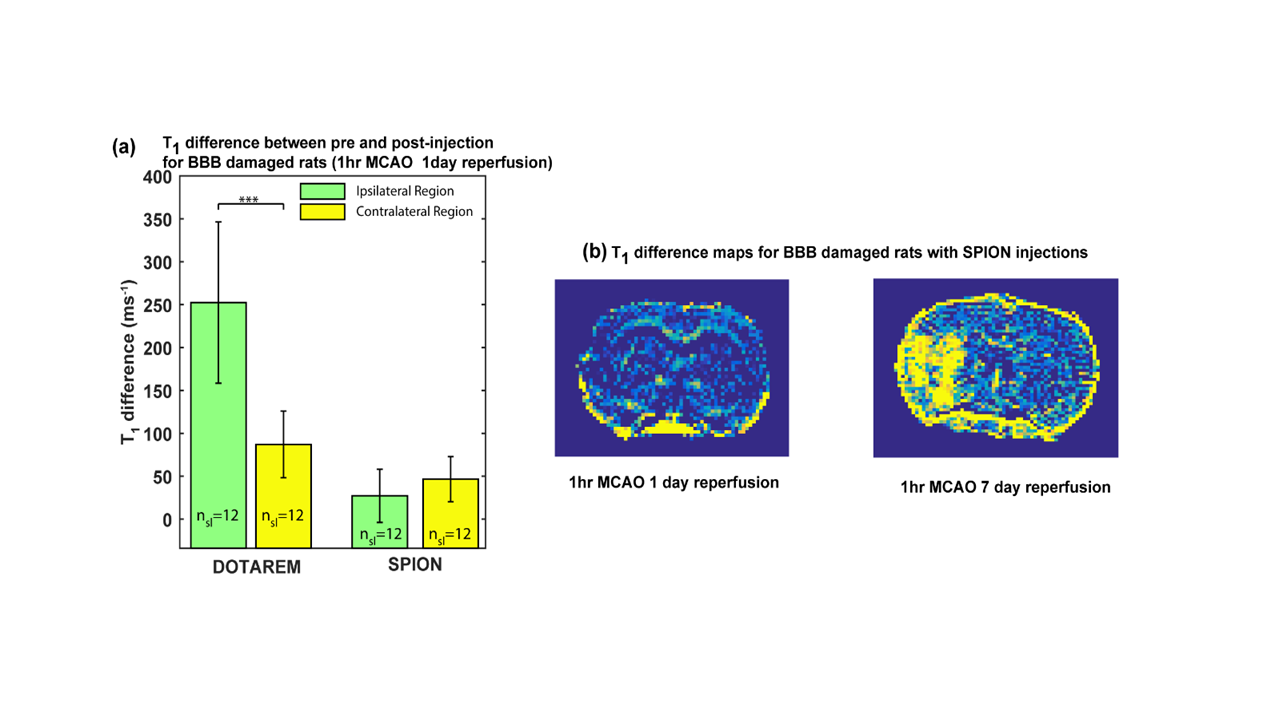

Supplement: S4 Fig — (a) The T1 difference before and after the CA injection for 1-hr MCAO and 1-day reperfusion model. The green and yellow bar graphs present the T1 difference of the ipsilateral infarction (ADC < 500 μm2/s) and contralateral regions, respectively. The statistical unit nsl is the number of slices. For the DOTAREM case (left), green bar: 252 ± 94 ms (nsl = 12), yellow bar: 87 ± 39 ms (nsl = 12), and p < 0.01 (p = 0.001). For the SPION case (right), green bar: 27 ± 31 ms (nsl = 12), yellow bar: 47 ± 26 ms (nsl = 12), and p > 0.05 (p = 0.229). (b) The T1 difference maps for 1-hr MCAO/1-day and 1-hr MCAO/7-day reperfusion models, respectively. Significant leakage of SPION is apparent in 1-hr MCAO/7-day reperfusion model. (TIF) [file pone.0201076.s004.tif]

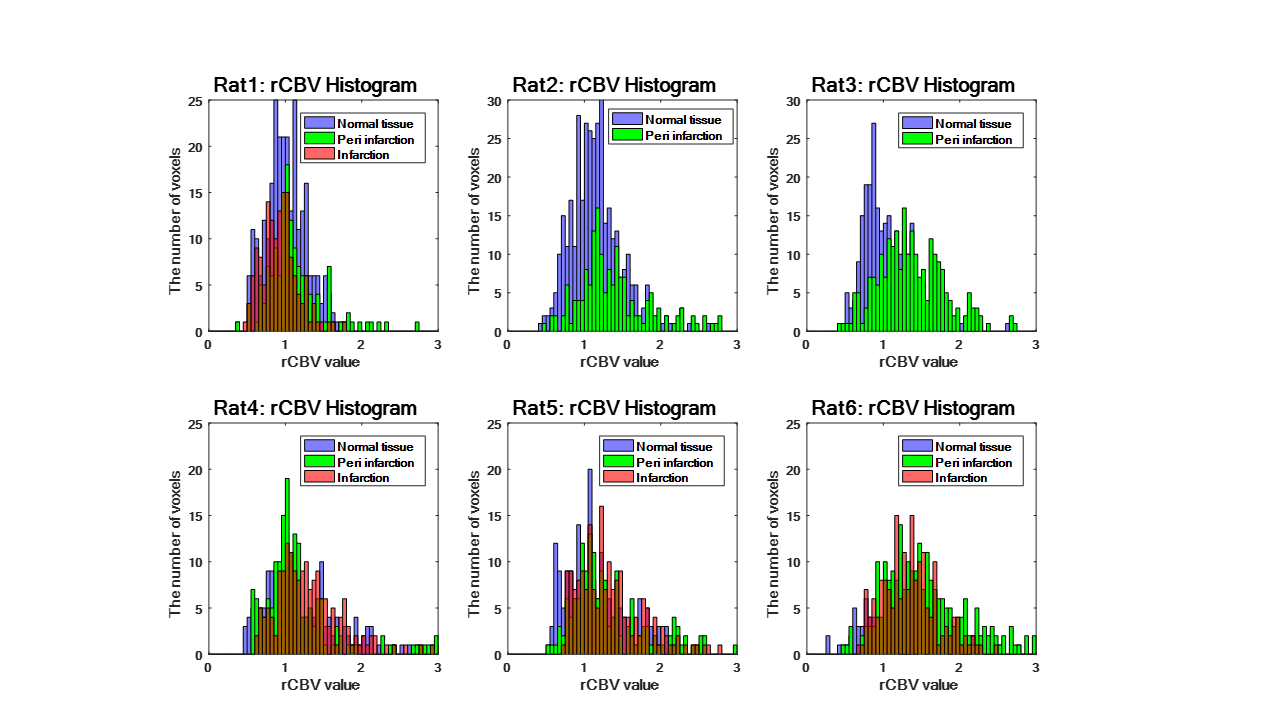

Supplement: S5 Fig — (TIF) [file pone.0201076.s005.tif]

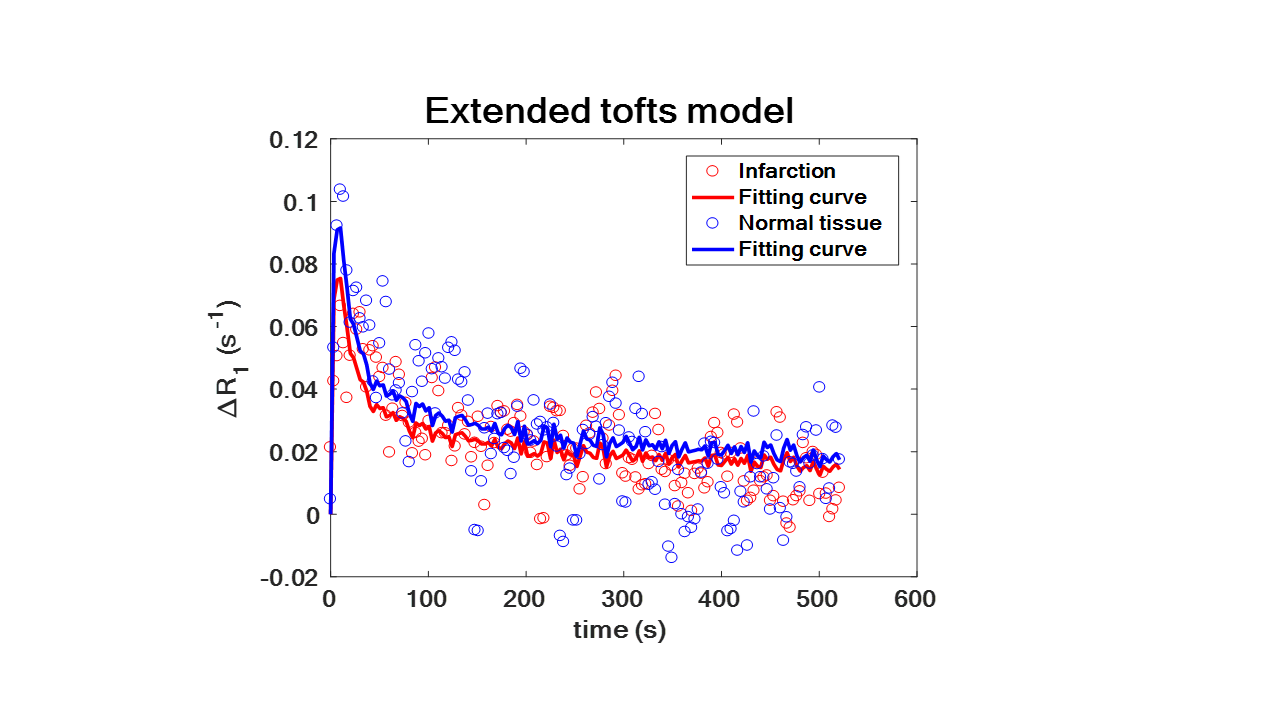

Supplement: S6 Fig — Blue and red dots represent time-signal data for normal and infarction regions, respectively. (TIF) [file pone.0201076.s006.tif]

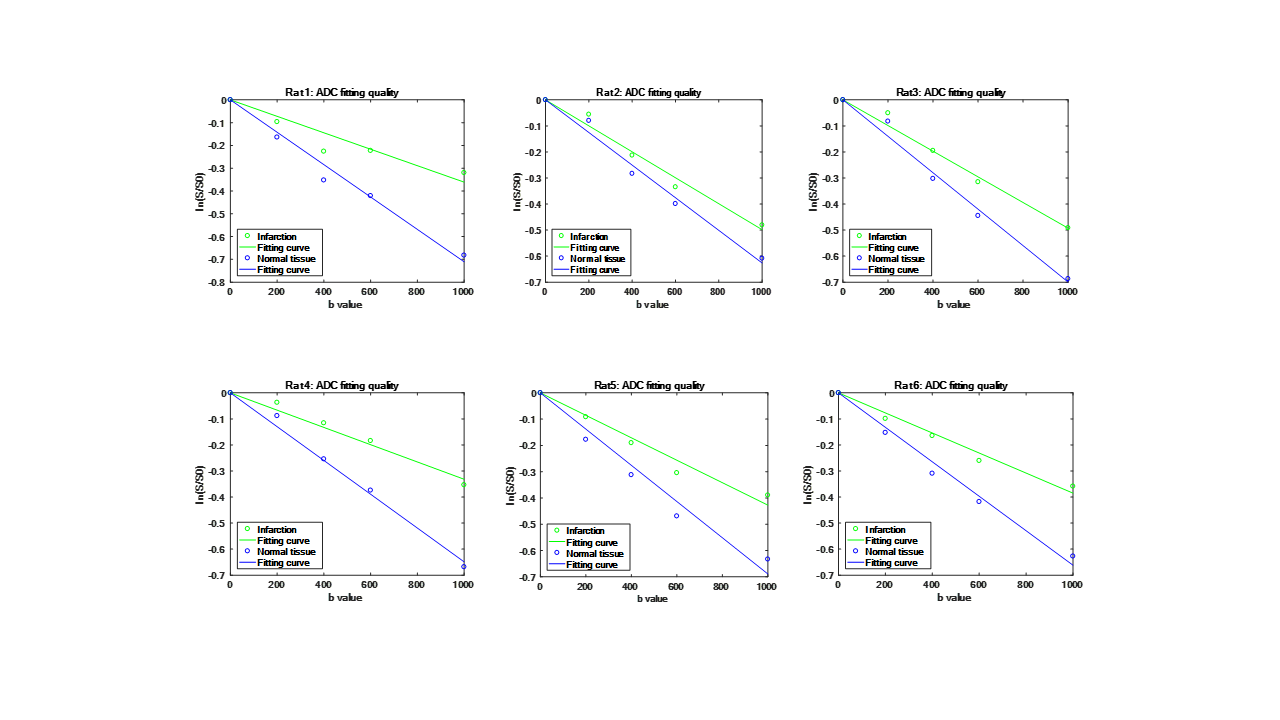

Supplement: S7 Fig — Blue and red dots represent diffusion data for normal and infarction regions, respectively for six stroke rats. (TIF) [file pone.0201076.s007.tif]

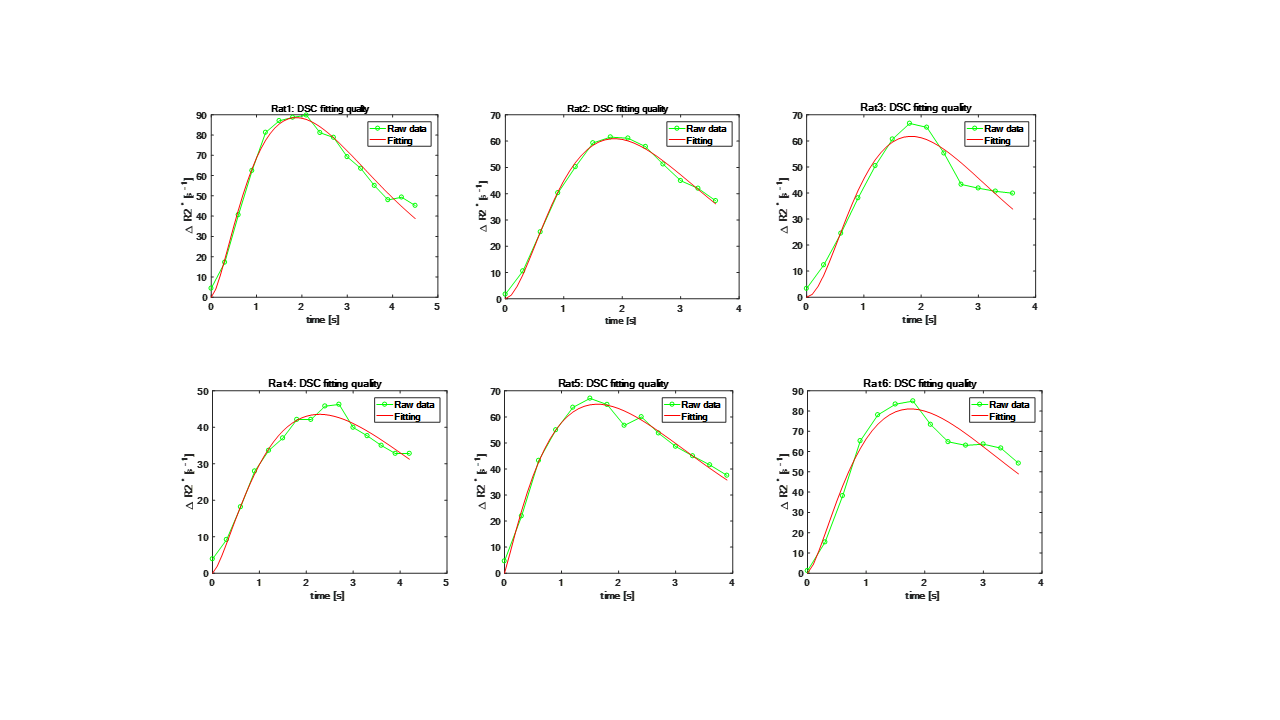

Supplement: S8 Fig — (TIF) [file pone.0201076.s008.tif]
